# Deletion of *CD163* Exon 7 Confers Resistance to Highly Pathogenic Porcine Reproductive and Respiratory Viruses on Pigs

**DOI:** 10.7150/ijbs.34269

**Published:** 2019-07-25

**Authors:** Haitao Wang, Liangcai Shen, Jingyao Chen, Xiaojuan Liu, Tan Tan, Yiqing Hu, Xiaofei Bai, Yuexin Li, Kegong Tian, Ning Li, Xiaoxiang Hu

**Affiliations:** 1 State Key Laboratory for Agrobiotechnology, College of Biological Sciences, China Agricultural University, Beijing, China.; 2 National Engineering Laboratory for Animal Breeding, China Agricultural University, Beijing, China.; 3 National Research Center for Veterinary Medicine, Luoyang, Henan Province, China.; 4 College of Animal Science and Veterinary Medicine, Henan Agricultural University, Zhengzhou, Henan Province, China.

**Keywords:** *CD163*, SRCR 5, Highly pathogenic PRRSV, CRISPR/Cas9, SCNT

## Abstract

Porcine reproductive and respiratory syndrome (PRRS) caused by PRRS virus (PRRSV) is a severe infectious disease in the swine industry. PRRSV infection is mediated by porcine CD163 (pCD163). Scavenger receptor cysteine-rich domain 5 coded by exon 7 of pCD163 is essential for PRRSV infection. In this study, we generated CD163 exon 7 deleted (CD163E7D) pigs using CRISPR/Cas9 mediated homologous recombination and somatic cell nuclear transfer (SCNT). The deletion of exon 7 had no adverse effects on CD163-associated functions. Pigs were further challenged with a highly pathogenic PRRSV (HP-PRRSV) strain. The CD163E7D pigs exhibited mild clinical symptoms and had decreased viral loads in blood. All CD163E7D pigs survived the viral challenge, while all the WT pigs displayed severe symptoms, and 2 out of 6 WT pigs died during the challenge. Our results demonstrated that *CD163* exon 7 deletion confers resistance to HP-PRRSV infection without impairing the biological functions of CD163.

## Introduction

Porcine Reproductive and Respiratory Syndrome (PRRS) has caused significant economic losses in pig farming. Since its first report in 1987 in the United States 1, PRRS had spread across the world for decades 2. The etiological agent of PRRS is Porcine Reproductive and Respiratory Syndrome Virus (PRRSV) 3, 4, which causes reproductive and respiratory symptoms in different ages of pigs, especially in piglets and sows. Once pigs are infected, fever, inappetence, lethargy, and breathing difficulty are observed in fattening pigs, and late-term abortion and fetal mummification frequently occur to sows. The newborn piglets delivered by infected sows are weak and suffer from severe respiratory symptoms 5-7. The large-scale reduction in pregnant sows and death of piglets caused a loss of more than 650 million dollars from 2005 to 2010 in the United States alone 8. In China, the epidemic of PRRS also leads to a devastating loss in the pig industry since its first emergence in 2006 9, 10.

PRRSV is a positive-stranded RNA virus belonging to the genus Arterivirus of the family Arteriviridae within the order Nidovirale 11, 12. Genetically, there are two different PRRSV genotypes: European (EU genotype, type 1) and North American (NA genotype, type 2). The approximate 15kb PRRSV genome contains 11 open reading frame and encodes at least seven structural proteins, including nucleocapsid protein N, protein M, and protein E, as well as GP2a, GP3, GP4, and GP5 13-15. PRRSV can only infect pigs, and the host cells are specific swine macrophage subsets, especially porcine alveolar macrophages (PAMs) 16. PRRSV can also infect other host cells *in vitro*, such as African green monkey kidney epithelial cell lines MA-104 and MARC-145 17.

The process of viral infection includes viral adhesion, host cells endocytosis, and genome release. Three cellular factors, heparin sulphate (HS) 18, CD163 19, and CD169 (Sn/cluster of differentiation 169) 20, involve in the binding, internalization, and uncoating of PRRSV respectively. Furthermore, studies have demonstrated that CD163 is the essential protein for PRRSV infection* in vivo* or* in vitro* 21-23.

CD163, also known as a hemoglobin/haptoglobin scavenger receptor or p155, belongs to the scavenger receptor cysteine-rich (SRCR) superfamily. CD163 was shown to bind the complex of hemoglobin and plasma protein haptoglobin (Hb-Hp) in early studies 24. Further studies demonstrated CD163 involves in PRRSV infection. The extracellular structure of CD163 contains nine SRCR domains, which are anchored on the cell surface by a transmembrane segment and a short cytoplasmic domain 25. The nine extracellular domains are separated by two proline-serine-threonine (PST) rich motifs located at the middle and the end of the extracellular region 26. Among the extracellular domains, SRCR domain 5 (SRCR5) is essential for PRRSV infection 27. Further structure-based mutation analysis revealed the important amino acid on SRCR5 for virus invasion *in vitro* 28.

Due to the high diversity of PRRSV, PRRS is difficult to control with traditional approaches 29, 30. However, gene editing of *CD163* made great progress in anti-PRRSV research. Although pigs with the complete ablation of* CD163* are resistant to PRRSV infection challenge *in vivo* 31, more studies focus on the genomic engineering of *CD163* exon 7, which encodes SRCR5, due to its essential role in PRRSV invasion, to avoid the potential harmful effects on CD163-associated biological functions. Pigs with *CD163* exon7 replaced with the* CD163L1* exon11 sequence (encoding human CD163L1 SRCR8) are resistant to type 1 PRRSV infection, but not to type 2 PRRSV infection 32. Pigs lacking SRCR5, which were generated using CRISPR/Cas9 editing in zygotes, are resistant to PRRSV-1 *in vivo* 33. However, it is unknown if the pigs lacking SRCR5 of CD163 are resistant to the type 2 HP-PRRSV *in vivo*. Here, we describe a different strategy, which used homologous recombination mediated by CRISPR/Cas9 and somatic cell nuclear transfer (SCNT), to generate *CD163* exon 7 deleted (CD163E7D) pigs. The generated CD163E7D pigs were challenged with high doses of HP-PRRSV and were remarkably resistant to PRRSV *in vivo*. The PRRSV-infected CD163E7D pigs also inhibited PRRSV infection to the WT pigs housed with them. We also showed the deletion of exon 7 had no adverse effects on other biological functions associated with *CD163 in vivo*.

## Materials and methods

### Ethics statement of Animal Usage

In this study, all application institutional the care and use of animals were followed. The animal experiments were performed according to the Guide for the Care and Use for Laboratory Animals issued by the Ministry of Science and Technology. The experimental protocols were approved by the Committee on the Ethics of Animal Experiments of China Agricultural University (Permit Numbers: CAU20170830-3, CAU20171215-3). The experimental animals were maintained in an animal facility in which room temperature of 20-26°C, humidity 40%-60%, and a 9/15 h light/dark cycle were maintained, and HEPA-filtered air was provided. The pigs were fed with a pig diet and tap water. At the end of the challenge, the surviving pigs were euthanized by ketamine.

### Vectors, Cells, and Viruses

The px330-501 vector was modified from the original pX330 vector (Addgene, plasmid #42230) in State Key Laboratory for Agrobiotechnology, China Agricultural University. The homologous recombination vector was constructed using pGKneo (provided by Dr. Chen Jinyao, China Agricultural University) as the backbone. The homologous arms containing the sequences of *CD163* were synthesized and inserted into pGKneo by restriction enzymes (New England Biolabs). Porcine fetal fibroblasts (PFFs) were obtained from 30-day-old Large White fetuses and maintained at 37°C with 5% CO_2_ in Dulbecco's modified Eagle's medium (DMEM) (Gibco, 11995-065) supplemented with 15% fetal bovine serum (FBS) (Gibco, 10099-141). The HP-PRRSV used in this study is strain JXA1 with GenBank Accession Number EF1122445, which was a generous gift of Professor Kegong Tian, National Research Center for Veterinary Medicine. Viral particles were prepared according to the method reported by Zhang et al. 34 and stored at -80°C until use. Marc145 cells were from the stocks in State Key Laboratory for Agrobiotechnology, China Agricultural University, and cultured with DMEM plus 5-10% FBS. The Marc145 cells were used for determining the viral titers of serum separated from the challenged pigs using the TCID_50_ method as reported by Zhang et al. 35.

### Cell transfection and selection

Electrotransfection of the vectors was applied according to the protocol provided by Amaxa Basic Nucleofector Kit (Lonza, VPI-1002). In brief, five micrograms of the px330-501 and homologous recombination vectors were mixed at a 1:2 ratio and added to 150μl electroporation medium. Approximate 1×10^4^ trypsinized PFFs were added to the medium cocktail, mixed, and transferred into a Lonza cuvette for electroporation. Then, all the cells were divided into multiple 10-cm plates with G418 (600 μg/mL, Promega) selection medium, and incubated for 9-12 days. The resistant clones were picked up with cloning cylinders. Healthy clones were sub-cultured and subject to genotyping. 30% sub-cultured cells from each colony were used for genomic DNA extraction. The forward primer E7F (5'-TTCTCCCTCACCGAAATGCT-3') and reverse primer E7R (5'-GCAGTGACGGAACAATCTCC-3') were used to amplify the exon7 fragment. The forward primer KZBF (5'-CTCCCAAGTGTCTTCCCTGATGCT-3') and reverse primer KZBR (5'-CAAGATGGATTGCACGCAGGTTCTC-3') were used to amplify a 7513bp fragment from the edited genome locus, which contains a part of the drug-selection marker, 5' arms, and the sequences before 5' arms in the WT genome. The primers designed for amplification of the 3' homological arm were KYBF (5'- GAGAACCTGCGTGCAATCCATCTTG-3') and KYBR (5'-TCTGACTCTGGTTCCCTGCAAACAG-3'). The expected PCR product size is 6020bp. Homozygous *CD163* exon7 null clones were expanded and cryopreserved for SCNT.

### Off-target analysis of sgRNA

Potential off-target sites (OTSs) were predicted on website http://crispr.genome-engineering.org/. The predicted OTSs were aligned to the whole pig genome on website http://asia.ensembl.org/index.html. The OTSs with less than 6 mismatches to the sgRNA sequence and located within a gene were picked up for further analysis. PCR amplification (primers were listed Table S1) and Sanger sequencing were performed to determine whether any point mutations exist.

### Generation of CD163E7D pigs by SCNT

The homozygous *CD163* exon 7 null cells were trypsinized and kept at 4°C as the donor cells. SCNT was performed as the previous study 36. In brief, cumulus-oocyte complexes (COCs) were aspirated and cultured in the maturation medium at 38.5°C for 40-44h. The mature COCs were digested with hyaluronidase (Sigma, H4273) for less than 5 minutes. The denudated oocytes that had released the polar bodies were enucleated by aspirating the cytoplasm adjacent to the first polar body with a 20μm glass pipette. Subsequently, a single donor cell was injected into the perivitelline space between the zona and cytoplasm of the enucleated oocytes. The reconstructed embryos were fused and activated with two successive dendritic cell pulses at 150V for 100ms using an electrofusion instrument (BLS, CF-150B). Then the embryos were cultured in porcine zygote medium at 38.5°C overnight and transferred into the oviduct of estrous recipient gilts. We monitored the pregnancy status of surrogates monthly until delivery. The piglets were delivered naturally. The ear skin of the newborn piglets was collected, and the genomic DNA was isolated for genotyping. The primers E7F/E7R were used to amplify the exon7 fragment. To detect whether the drug-selection gene was excised in the pigs, the forward primer 169F (5'-TGAATTGCCTCTCAGTCTG-3') and reverse primer 172R (5'-CATGATAGGAGTAAGCCAG-3') were used to amplify the whole fragment between homologous arms. Next, total RNA in the lungs of piglets was isolated with TRIzol (Invitrogen, 15596026) for RT-PCR. Primers ZLBF (5'-TCAGTGCCTGCTTGGTCACTAG-3') and ZLBR (5'-CCGTTCATCTGCTTTCAGGCAAG-3') were used to amplify *CD163* transcript. The tissue samples of stillborn piglets were collected for further analysis.

### Western blot

Tissues were collected and lysed by immunoprecipitation (IP) lysis buffer (Biyotime, P0013). Protein concentrations were quantified with a BCA kit (Beyotime, P0010). An equal amount of total protein from each sample was separated on 10% sodium dodecyl-sulphate polyacrylamide gel electrophoresis (SDS-PAGE) and transferred to a polyvinylidene difluoride membrane (Millipore). Then membranes were blocked with 5% non-fat milk and incubated with a mouse anti-pig CD163 monoclonal antibody as the primary antibody. After washing with TBST, HRP-conjugated anti-mouse IgG was used as the secondary antibody. β-actin was blotted as a loading control. Pierce™ ECL Western Blotting Substrate (Thermo Fisher Scientific) was used to visualize the signals, and the images were captured with a camera (Tanon, 5200).

### ELISA

PRRSV-specific antibody was detected using IDEXX PRRS X3 Ab Test (IDEXX Laboratories) according to the protocol of manufacturer. Serum samples were considered positive for PRRSV antibody if the S/P ratio was greater than 0.4. The amount of Hp in serum was measured using a pig ELISA kit (Cloud-Clone Crop Inc.) according to the manufacturer's instructions. Each sample was detected in triplicate.

### Viral Challenge

The *in vivo* infection of the experimental pigs with HP-PRRSV was performed at National Research Center for Veterinary Medicine in Luoyang, Henan province. All the tested pigs were female, 8 weeks old, and were transferred to the challenge facility to acclimate 5 days before the challenge. Pigs were divided into four groups in two separate rooms: the challenged CD163E7D pigs (n=6) and 6 non-challenged WT pigs were maintained in one room, while the challenged WT pigs (n=6) and another 6 non-challenged WT pigs were housed in the other room. Before the challenge, the pigs were confirmed to be negative for PRRSV antibody in the serum and free of PRRSV infection. The rectal temperatures of all the pigs were normal, and there were no any observable clinical symptoms, and all the pigs were also energetic and ate actively. The pigs were challenged with the HP-PRRSV strain JXA1 by intramuscular injection of 10^6.5^ TCID_50_ of viruses per animal. During the intramuscular injection, the unchallenged pigs that would be housed with the challenged pigs were separated from the challenged pigs temporally and transferred back several hours later. To avoid the leakage of virus from the needles, they were left to stay in the muscle for seconds after injection, alcohol wipes were immediately used for disinfection and were hold for several minutes at the injection sites after pulling out of the needles. Infected pigs were monitored for the next 21 days. The rectal temperature was measured twice daily, and clinical symptoms including respiratory distress, lameness, diarrhea, inappetence, and fever were recorded and scored as previously described 37, 38. In short, the pigs were scored 0 for normal, 1 for mild inappetence or decreased demeanor, 2 for sneezing/coughing, diarrhea, or rubefaction, 4 for severe respiratory distress, lameness, blue ears, or stopping feed intake. Blood samples were collected on days 0, 3, 5, 7, 10, 14, 17, 21 and allowed to clot. The serum was separated and preserved at -80 °C. The surviving pigs were sacrificed by euthanasia and their tissues were collected for necropsy.

### Immunohistochemistry Staining

Tissues, including lung, tonsil, spleen, lymph node of the different challenged groups, were collected and subject to immunohistochemistry to detect PRRSV as described previously 39. The primary antibody was a mouse monoclonal antibody against PRRSV nucleocapsid protein. The slides were visualized using a microscope.

## Results

### Generation of *CD163* exon 7 deleted PFFs

**Fig 1:**
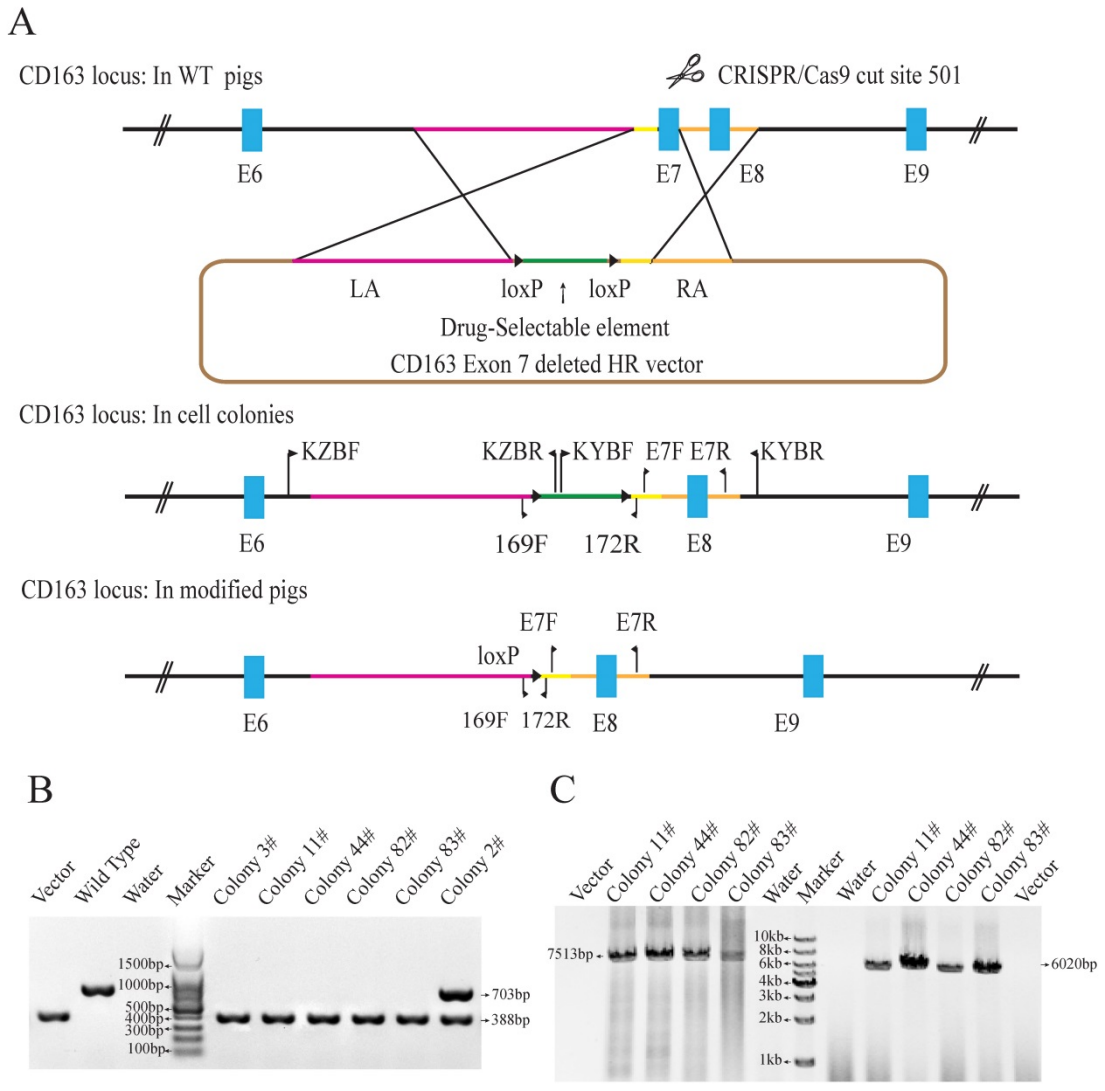
** Generation of *CD163* exon 7 deleted PFFs.** (**A**) Strategy for generation of *CD163* exon 7 deleted allele. The WT *CD163* exon 7 locus and donor vector are shown. The donor vector is composed of two homologous arms (pink and amber lines) and a drug-selection element (green line) flanked by two loxP sites (black triangles). The Cas9 enzyme makes a double-stranded break at the site targeted by 501-sgRNA. After homologous recombination repair mediated by the homologous arms, *CD163* exon 7 will be replaced by the drug-selection cassette, resulting in an exon 7 deleted allele. The excision of the drug-selection gene is mediated by the Oct4-Cre element during the early stage of embryonic development, and a loxP sequence will be left in intron 6 of *CD163.* (**B**) Identification of exon 7 deletion in PFFs. E7F/E7R primers were used to amplify PFF colony DNA. (**C**) PCR amplification of the two homologous arms. The PCR products for the left (left) and right arms (right) are expected to be 7513bp and 6020bp in size, respectively. The result from the control homologous recombination vector was negative.

To achieve *CD163* exon7 biallelic deletions in PFFs, the CD163E7D homologous recombination vector was constructed as a donor plasmid. The donor vector was constructed by modifying the pKGneo vector and contains two homologous arms. A drug-selection cassette and an Oct4-Cre element were flanked by two loxP sites between the arms for excision of the drug-selection gene. A DTA (diphtheria toxin A-chain) element was put outside the homologous arm to avoid random integration. The donor vector was used as a template to repair the DNA double-strand break generated by sgRNA/Cas9 via homologous recombination (Fig. 1A). The 6255bp left arm and 999bp right arm are located upstream and downstream of exon 7, respectively. The left arm was cloned into the Sac II and Not I sites of pGKneo vector, and the right arm was cloned to the Sal I and Asc I sites of pGKneo vector.

The Cas9 vector pX330-501 was mixed with the donor vector and transfected into porcine fetal fibroblasts. 62 colonies were screened using three pairs of primers to detect the recombination events. Primers E7F/R, which amplify the exon 7 sequences, were used to determine whether the exon 7 was deleted. Primers KZF/R and KYF/R were used to detect the recombination event by amplifying the fragments spanning the homologous arms and their adjacent genomic sequences. Our results showed that E7F/R primers amplified a 388bp product from the CD163 E7 deleted allele and a 703bp from the WT allele (Fig. 1B). KZF/R primers amplified a fragment of 7513bp containing the 5' homologous recombination arm and the drug-selection marker, and KYF/R primers amplified a fragment of 6020bp containing the right homologous arm (Fig. 1C). After PCR amplification and Sanger sequencing analysis of the products, a total of 5 colonies were demonstrated to carry the *CD163* exon7 biallelic deletions.

Due to RNA-based recognition, the off-target effect of CRISPR/Cas9 system must be concerned. To check whether off-target genome modifications occurred, 7 potential off-target sites, which have less than 6 mismatches with the guide RNA sequence, were determined and screened (Table 1). All the seven possible off-target sites were examined by PCR and Sanger sequencing. The results showed no off-target mutations occurred in the genomes of all the 5 colonies. The results of PCR and Sanger sequencing of colony 11# were showed in Fig. S1. Two of the CD163E7D colonies were used as donor cells for SCNT.

### Generation of *CD163* exon7 deleted pigs

**Fig 2:**
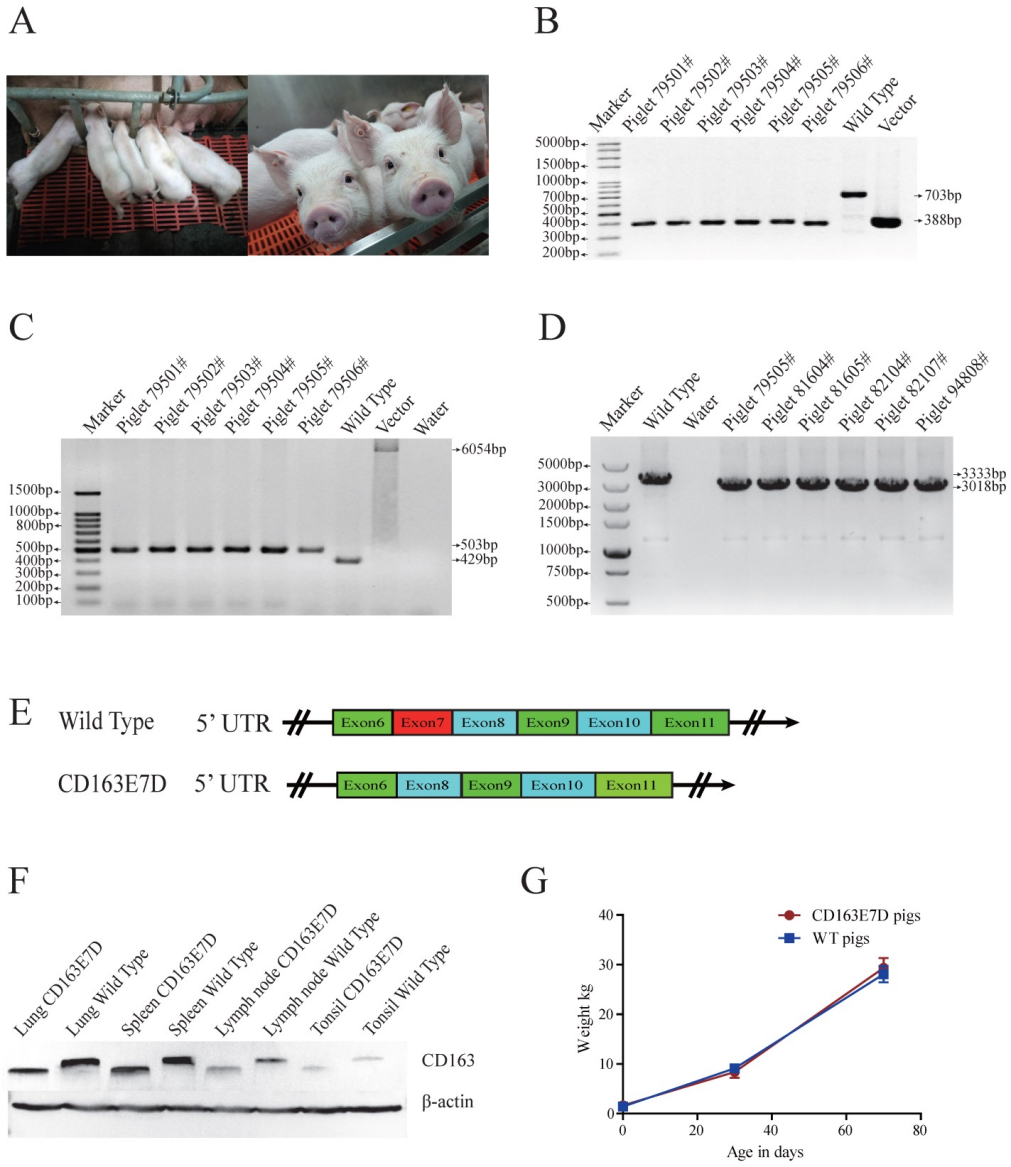
** Generation of *CD163* exon7 deleted pigs via SCNT.** (**A**) Represented pictures of 10-day-old (left) and 8-week-old (right) CD163E7D piglets. (**B**) Genotyping of the genome-modified pigs (#79501-#79506) by PCR using primers E7F/R. (**C**) PCR products of primers 169F/172R demonstrated the absence of the drug-selection gene in the pigs. The PCR product from CD163E7D pigs was 503bp, and the products of the control homologous recombination vectors were 6054bp. (**D**) RT-PCR proved that the transcripts of *CD163* in the lung of the CD163E7D and WT pigs were 3018bp and 3333bp, respectively. (**E**) A schematic view of the CD163 transcripts in CD163E7D and WT pigs. (**F**) Western blot shows that the CD163E7D pigs express a smaller CD163 protein in the tissues compared with the WT pigs. (**G**) Growth curves of the CD163E7D pigs (n=4, 94801#-94804#) and WT pigs (n=15).

The biallelic mutant cells derived from colonies 11# and 44# were chosen as donor cells for SCNT. Cells from the single colony or the pool of these two colonies were used for nuclear transfer. A total of 2192 reconstructed embryos were transferred to 6 surrogate mothers, and four surrogate sows were successfully pregnant. 26 piglets were delivered naturally (Table 2, Fig. 2A), including 4 stillborn piglets, 2 weak piglets died in 15 days, and 20 healthy piglets. The same PCR primers used for detecting the exon 7 deletion in PFFs were used for genotyping the piglets (Fig. 2B), and the exon 7 deletion was confirmed in the cloned animals. As the Oct4-Cre element was designed in the vector for the excision of the drug-selection gene in the early stage of embryonic development, primers 169F/172R were used for amplification of the whole fragment between homologous arms. As expected, PCR products of CD163E7D pigs were 502bp (Fig. 2C), and Sanger sequencing analysis showed only one loxP sequence was left in intron 6 of *CD163*, confirming the drug-selection gene was excised in the pigs. Furthermore, to detect the expression of* CD163*, the piglet lung RNA was extracted for RT-PCR. Primers ZLBF/R were used to amplify *CD163* cDNA. The Sanger sequencing results of the PCR products showed the exon 7 of *CD163* cDNA had been deleted, and the transcript of CD163E7D pigs was 315bp shorter than the WT transcript (Fig. 2D and E). The *CD163* cDNA sequence of CD163E7D pigs and its predicted amino acid sequence were showed in Fig. S2. As a result, Western blot showed that the CD163 protein of CD163E7D pigs was 13.4kDa smaller than that of WT pigs in the lungs, tonsils, spleens, and lymph nodes (Fig. 2F). The weights of CD163E7D pigs and WT pigs were recorded, and the growth curves showed the CD163E7D pigs had similar growth rate as the WT pigs (Fig. 2G). There were no significant differences in weights between the 2 groups on their birthdays, 30 days, and 70 days. These results demonstrated that the CD163E7D pigs expressed CD163 only lacking SRCR5, and the CD163E7D pigs grew normally.

### *CD163* exon 7 deletion does not influence its normal biological functions

**Fig 3:**
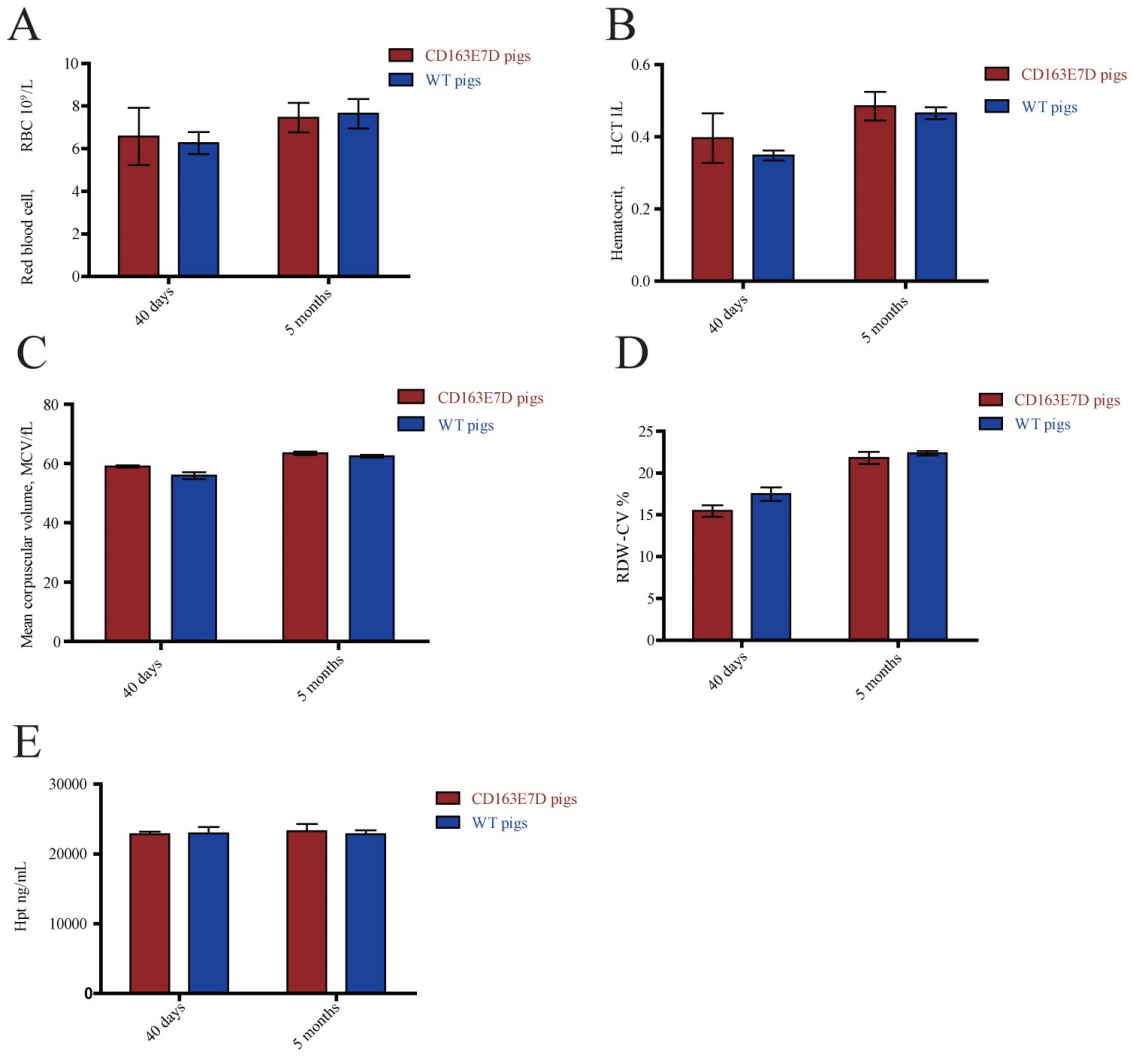
***CD163* exon 7 deletion does not influence its normal biological functions.** The blood samples of CD163E7D (n=5) and WT pigs (n=5) were collected for the test at the ages of 40 days and 5 months. (**A**) The red blood counts of the CD163E7D and WT pigs. (**B**) The hematocrit level of the CD163E7D and WT pigs. (**C**) The mean corpuscular volumes of the CD163E7D and WT pigs. (**D**) The red blood cell distribution width of the CD163E7D and WT pigs. (**E**) The serum Hp concentrations of the CD163E7D and WT pigs. All data are presented as the mean±SD.

The biological functions of WT CD163 include prompting erythroblast growth and mediating the removal of hemoglobin from the blood. To determine whether the deletion of exon 7 influenced its normal functions, blood samples of 6-week-old and 5-month-old pigs were collected and subjected to routine blood tests. As shown in Fig. 3A, B, C and D, no significant differences were observed in red blood cell counts, blood hematocrit levels, red blood cell distribution width, or mean corpuscular volumes between the WT and CD163E7D pigs. Moreover, free hemoglobin binds to haptoglobin to form a Hb-Hp complex, which interacts with the SRCR3 domain of CD163. Thus, the serum of above pigs was separated to detect the Hp levels by ELISA (Fig. 3E). The results showed there were no differences in Hp levels between the WT and CD163E7D pigs. These results demonstrated that the* CD163* exon 7 deletion had no adverse effects on its normal biological functions.

### *CD163* exon7 deleted pigs are resistant to HP-PRRSV

To test whether the CD163 exon 7 deletion can protect pigs from the viral challenge *in vivo*, 8-week old CD163E7D pigs and WT Large White pigs were challenged with a highly pathogenic PRRSV strain, JXA1 (Table S2). The serum of all the tested pigs was negative for protein N and PRRSV-specific antibody before the viral challenge (Fig. S3 and S4). The viral challenge had lasted for 21 days, and clinical characteristics, including rectal temperatures, overall body condition, and respiratory disorders, were recorded daily until the animal death or the end of the challenge.

**Fig 4:**
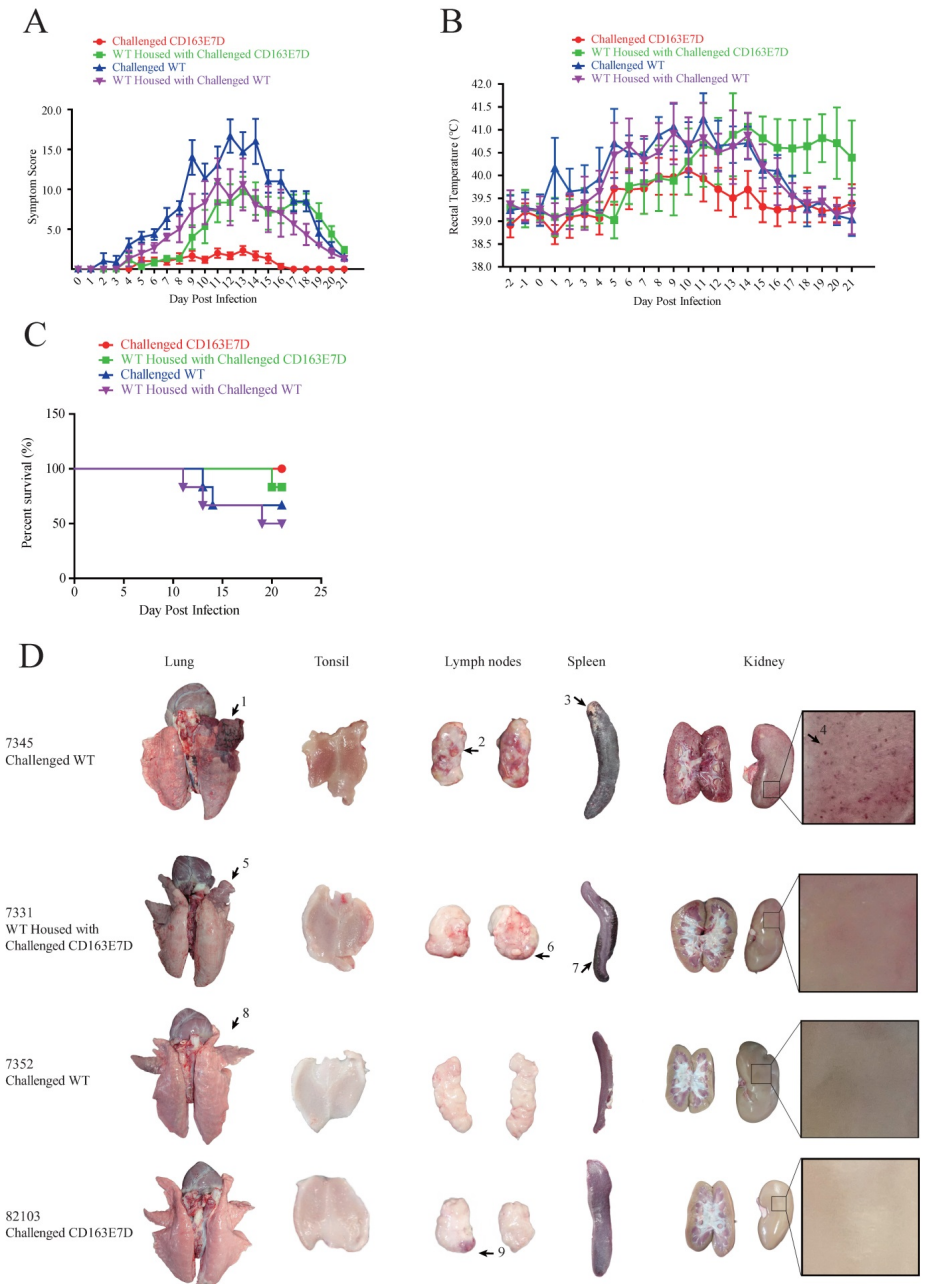
***CD163* exon 7 deleted pigs are resistant to HP-PRRSV.** (**A**) Symptom scores of the challenged WT and CD163E7D pigs, as well as the pigs housed with them, during the viral challenge. (**B**) Rectal temperature curves of the tested pigs during the viral challenge. (**C**) Survival curves of the tested pigs. (**D**) Macroscopic lesion of lungs, tonsils, spleen, lymph nodes, and kidney of the pigs of four severity categories (see **Table 3**). Only representative pictures were shown. Arrow1 indicates a lung hemorrhage and parenchymal lesion with secondary bacterial infection; arrows 2, 6, and 9 indicate lesions of lymph node hemorrhage; arrow 3 indicates a lesion of spleen infraction with secondary bacterial; arrow 4 indicates a lesion of kidney with blood spot; arrows 5 and 8 indicate lung hemorrhage lesions; arrow 7 indicates a lesion of spleen infraction.

During the course of the disease, WT pigs started to show mild symptoms, such as fever and sternutation, at 2-5 days post inoculation (DPI). Then the signs continued and worsened gradually: the pigs exhibited respiratory disorders, lameness, diarrhea, and inappetence at 10-14 DPI. The most severe pigs presented with the blueing of ears and extremities, respiratory failure, ataxia, muscle paralysis, nystagmus, and other neurological symptoms. Some of the WT pigs died at this time, and other pigs recuperated and survived, but still exhibited some symptoms. Symptom scores were used to evaluate the disease course. The symptom scores of the challenged WT pigs and the pigs housed with them arose the earliest at 2 DPI, while the CD163E7D pigs only showed very mild symptoms at the later time. The symptoms of WT pigs became the most severe at 12 DPI, and some WT pigs started to die at 11 DPI (Fig. 4A). The surviving WT pigs still had mild symptoms at the end of the challenge. However, the mild signs of CD163E7D pigs disappeared at 16 DPI, and all the pigs were healthy without relapse at the end of the challenge (Fig. 4A). The WT pigs housed with the challenged CD163E7D pigs started to show symptoms the latest (Fig. 4A). One of them died at 20 DPI, and the others started to recover before the end of the challenge (Fig. 4A). The rectal temperature changes were similar to the symptom scores in all the groups of pigs. The challenged WT pigs and the pigs housed together with them had a fever as early as 2-4 DPI. Their rectal temperature increased to 41°C at 10-14 DPI. The surviving pigs recovered from fever at the end of the challenge. The challenged CD163E7D pigs had fevers later than WT pigs at 5 DPI, and their rectal temperatures were never higher than 41℃ during the entire process. The housed pigs had fever the latest, and they showed most severe fever between 13-19 DPI (Fig. 4B). At the same time, the surviving challenged WT pigs and their housed pigs had recovered from high fever. The results showed the symptoms and fever had been postponed, but not more severe in the WT pigs housed with the challenged CD163E7D pigs.

All the CD163E7D pigs survived the viral challenge, while 66.6% challenged WT pigs survived. The WT pigs housed with the challenged CD163E7D pigs had an 83.3% survival rate, while the survival rate of non-challenged WT pigs housed with challenged WT pigs was only 50% (Fig. 4C).

### There were much less macroscopic lesions in CD163E7D pigs

After the viral challenge, the animals were euthanized, and the macroscopic lesions and pathology of their organs were examined. The CD163E7D pigs had almost no visible lesions in the tissues and organs. Only a slight lymph node hemorrhage was observed in CD163E7D pig #82103 (Fig. 4D), which showed the most severe symptoms in this group, other CD163E7D pigs had no visible lesions. WT pigs in other groups showed more serious lesions. Pulmonary parenchymal lesions, lung edema, lung hemarrhage, spleen infarct, renal hemorrhage, lymphatic hemorrhage, and secondary bacterial infection were common among the dead WT pigs. The detailed lesions in different treatment groups were listed in Table S3. The WT pigs housed with the challenged CD163E7D pigs had fewer lesions than those housed with challenged WT pigs (Table 3).

### The CD163E7D pigs had less viral infection

**Fig 5:**
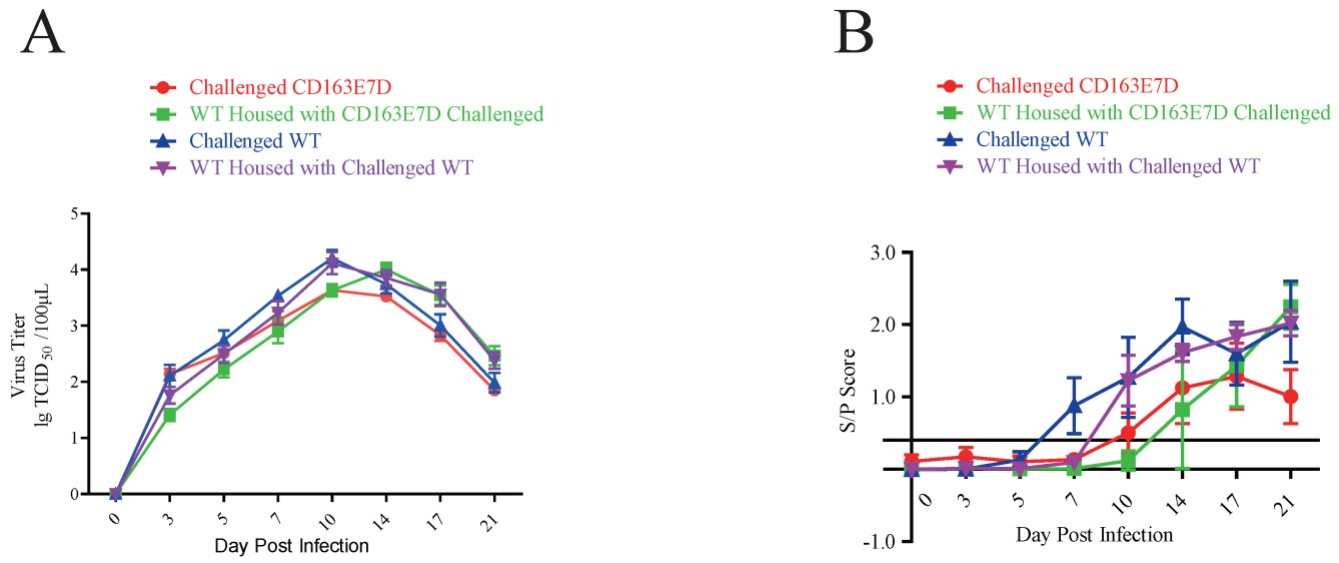
** The CD163E7D pigs had less viral infection.** (**A**) Serum virus loads of all tested pigs determined by TCID_50_ assay. (B) The amounts of serum antibody against PRRSV nucleocapsid (N) protein of the tested pigs during the challenge.

Serum PRRSV viremia of the tested pigs was determined by TCID_50_ assay using Marc45 cells (Fig. 5A). A similar result to the symptom scores was observed. For WT pigs, the mean viremia titer increased to the peak steadily at 10-14 DPI after the challenge. However, the blood viral titers of CD163E7D pigs, which were never higher than the challenged WT pigs, increased more slowly and declined to a low level eventually. The rise of blood viral titer was delayed in the 2 non-challenged WT groups housed with either the challenged WT or CD163E7D pigs. The serum antibody levels were detected with ELISA. As a result, the challenged CD163E7D pigs had a lower PRRSV protein N antibody level than WT pigs, and the antibody levels of non-challenged pigs housed with challenged pigs were delayed compared with their challenged counterparts (Fig. 5B). Immunohistochemistry of PRRSV nucleocapsid protein also showed similar results: the viral protein signals were stronger and more intense in the lungs, spleens, tonsils and brains of most WT pigs, but positive signals were only observed in the tonsil of one CD163E7D pig (Table 4, Fig. S5).

## Discussion

In this study, CD163 exon 7 precisely deleted pigs were generated with CRISPR/Cas9 mediated homologous recombination and somatic cells nuclear transfer. The genome editing of CD163 had no observable influences on the normal biological functions associated with the gene, such as erythroblast growth and Hb-Hp complex clearance. When the pigs were challenged with HP-PRRSV *in vivo*, the clinical signs appeared mild and lasted shortly in the CD163E7D pigs compared with the WT pigs. The WT pigs housed with the challenged CD163E7D pigs exhibited symptoms the latest and had a higher survival rate than the pigs housed with the challenged WT ones. Our results suggest that the CD163E7D pigs are resistant to HP-PRRSV infection without influencing its normal functions.

Genome editing of *CD163* is not the only way to prevent PRRSV infection. However, traditional approaches based on vaccines or medicines were difficult to control PRRSV due to the diversity of the virus. Early studies primarily aimed at preventing PRRSV replication in cells to protect pigs against PRRSV. A study used RNA interference to silence viral gene expression in order to inhibit PRRSV replication* in vitro* 40. Another study showed that adenoviruses carrying short-hairpin RNAs (shRNAs) against PRRSV genome could deliver the shRNA efficiently and inhibit PRRSV from spreading *in vitro* and *in vivo* 41. PRRSV duplication was reduced in transgenic F1 pigs expressing PRRSV-specific siRNA compared with none-transgenic pigs, demonstrating the consistency of the RNAi-based approaches 42.

Different from preventing PRRSV replication, the genome-editing approach targets the PRRSV infection process mediated by the cell surface receptors of viral hosts. As an interacting protein of PRRSV, CD169 was considered to be an important determinant for PAM permissiveness of PRRSV infection. However, knockout of *CD169* had not increased the pigs' resistance to PRRSV, and the *CD169^-/-^* pigs had the same disease course as the WT pigs after viral challenge 43. In contrast, *CD163*-modified pigs from different origins were prominently resistant to genotype 1 and 2 PRRSVs 21. CD163 consists of nine SRCR domains, and SRCR 5 was considered to be essential for PRRSV infection. To avoid the impairment of CD163 normal biological functions, genomic modifications of SRCR5 only are a better strategy than the knockout of entire CD163 gene. Thus far, substitution of SRCR5 with a homologous domain, missense point mutations in SRCR5, and knockout of CD163 SRCR 5 were used to obtain *CD163* gene-editing pigs.

Recent studies showed that pigs in which SRCR5 was substituted with human homologous CD163L1 SRCR8 were resistant to the infection of genotype 1 PRRSV, but the animals were still permissive to the infection of genotype 2 viruses 32. In Burkard's studies, SRCR 5 of CD163 was knocked out using two sgRNAs flanking exon 7 of CD163 in pig zygotes. presented resistance to both type 1 and type 2 PRRSV *in vitro* 44. In their latest study, the CD163 SRCR5 null pigs were conferred resistant to the type 1 PRRSV, but the animal's resistance to type 2 viruses was not tested 33. Using more sgRNAs for gene editing increased the possibility of off-targeting, and microinjection of sgRNAs might result in mosaicism. In the present study, we used CRISPR/Cas9 mediated homologous recombination to edit the *CD163* exon 7 locus in porcine fetal fibroblasts and further used nuclear transfer to generate homozygous *CD163* exon 7 deleted pigs. No off-target mutations were detected in the genome of the donor colonies. The viral challenge of the CD163E7D pigs with HP-PRRSV showed the pigs are resistant to the viruses.

As a cell surface marker, CD163 was not only identified as a receptor of viruses, such as PRRSV and ASFV 45, but also involved in erythroblast adhesion, clearing of the Hb-Hp complexes, and potential anti-inflammatory activity 24. The precise deletion of SRCR5 in pigs may have advantages over the CD163 knockout animals described previously, which were randomly introduced a premature stop codon in the exon 3 21 or exon 7 31. The CD163 SRCR 3 domain plays a vital role in clearance of Hb-Hp complexes 46, and the SRCR 2 domain is an adhesion receptor for erythroblasts 47. Thus, it is necessary to examine the functions of CD163 in SRCR5 null pigs. Our results showed the exon 7 deletion does not influence the normal functions of CD163. The effects of exon 7 deletion on animal growth and reproductivity need to be evaluated in future studies.

In the viral challenge experiment, although the symptoms of CD163E7D pigs (only transient fever were observed) were mild, the CD163E7D pigs were still infected by PRRSV as shown by the viremia titers. This observation is different from Burkard's study 33, in which no PRRSV infection was observed. We speculate that the different PRRSV strains used in the 2 studies are the primary reason. In Burkard's study, the subtype strains of PRRSV-1 BOR-57 were inoculated intranasally into the pigs. During the challenge, no respiratory or other severe symptoms were observed except decreased demeanor in only one WT pig. The fever of WT pigs lasted shorter than our study, and no pigs died in the challenge. However, in our study, the pigs were inoculated with type 2 HP-PRRSV by intramuscular injection. This strain was isolated from the infected pigs in China in 2006, and its nsp2 region had a unique 30-amino acid deletion. The WT pigs had sustained fever, respiratory symptoms, and behavioral abnormalities, and some pigs died because of the disease. Differences among the viral strains in infectivity are a possible reason for the various clinical signs. Moreover, in our previous study, SRCR5-substituted pigs were also generated and challenged with HP-PRRSV strains 48. The results showed the PAMs from the modified *CD163* were completely protected from the viral infection *in vitro*, but the pigs showed a transient viremia after the infection, and one pig died in the viral challenge. *In vivo* responses of the animals are more complicated than the cultured cells *in vitro.* Other types of monocytes/macrophages cells, such as bone marrow-derived macrophages, pulmonary interstitial or splenic macrophages, may also be infected by PRRSV* in vivo* 49. Other unknown reasons may exist and need further studies. In our study, these cell populations and other factors may have contributed to the lower infection in the CD163E7D pigs, which requires further studies.

Furthermore, we also set up two pig groups that were housed with either the challenged WT pigs or the challenged CD173E7D pigs. Although one died at the end of challenge experiment, the WT pigs housed with the challenged CD163E7D pigs showed delayed and milder clinical signs, and the survival rate of this group was also higher compared with the WT pigs housed with the challenged WT pigs. We speculate that the failure of PRRSV infection into the host cells of the CD163E7D pigs reduced virus replication and inhibited viral spreading. The result demonstrates that the CD163E7D pigs can effectively suppress infection of PRRSV in a herd.

In conclusion, we generated *CD163* exon 7 deleted pigs by CRISPR/Cas9 mediated homologous recombination and SCNT. In the viral challenge experiment of HP-PRRSV, the CD163E7D pigs had less viral infection and recovered rapidly and were able to reduce the mortality in the herd. Meanwhile, the deletion of *CD163* exon 7 does not impair the biological functions associated with* CD163*.

## Supplementary Material

Supplementary figures and tables.

## Figures and Tables

**Table 1:** Off-target analysis of colony #44.

	Sequences^a,b^	Mism-atches	Gene	Position in genome	Results^c^
Target site	GGAACTACAGTGCGGCACTGTGG	-	CD163	-	-
Off site1	GGGCCTGCATTGCGGCACTGAGG	4MMs	SLC17A5	chr1:92337898-92337920	no off-target occurred
Off site2	GGGGCTGCAGGGCGGCACTGAGG	4MMs	KCNK12	chr3:92999538-92999560	no off-target occurred
Off site3	AGAACCACAGTGCGACACTGAGG	3MMs	ANKH	chr16:4129263-4129285	no off-target occurred
Off site4	GAAACGACAGTGCGACACTGAGG	3MMs	APBA2	chr1:144181060-144181082	no off-target occurred
Off site5	GGACGTGCAGTGAGGCACTGTGG	4MMs	MEGF11	chr1:164139020-164139042	no off-target occurred
Off Site6	TGAATACAAGTGCGGCACTGGGG	5MMs	ACVR2A	chr15:4249480-4249502	no off-target occurred
Off site7	GAAGTCCCTGTGCGGCACTGTGG	6MMs	DNAJC3	chr11:65260383-65260405	no off-target occurred

^a^ The nucleotides mismatched with 501-sgRNA were labeled red. ^b^ The PAMs were labeled blue. ^c^ No mutations were detected at these sites.

**Table 2:** Somatic cell nuclear transfer results of CD163 exon7 deleted pig.

Recipient ID	Donor colonies	No. of embryos transferred	Day of estrus	Piglets status
20303	11#	390	1	5 live piglets, 1 dead piglets
10603	11#	400	1	No piglet
21007	11# , 44#	310	1	4 live piglets (one died at day 5), 1 dead piglet
39003	11# , 44#	396	1	6 live piglets (one died at day 13), 1 dead piglet
10112	11# , 44#	346	1	No piglet
33003	44#	350	1	7 live piglets, 1 dead piglet

**Table 3:** Macroscopic lesions of the challenged pigs and the pigs housed with them^a^.

Groups	Lung hemorrhage	lung edema	Pulmonary parenchymal lesion	spleen infract	kidney with blood spot	secondary bacterial infection	lymph node hemorrhage	tonsil hemorrhage	Status before euthanasia
Challenged CD163E7D	0	0	0	0	0	0	1	0	All survived
WT housed with challenged CD163E7D	1	1	6	4	1	1	6	1	1 died, 5 survived
Challenged WT	2	2	5	3	2	1	6	4	2 died, 4 survived
WT housed with challenged WT	3	3	6	3	3	1	6	3	3 died, 3 survived

^a^ The numbers of pigs that had the lesion in the group (n=6) were listed in the columns.

**Table 4:** Results of PRRSV immunohistochemistry staining.

Group	Pig ID	Tonsil^a,b^	Lung	Lymph Node	Spleen	Kidney	Brain
Challenged WT	7345 (Died at 13 dpi)	+	+	+	-	-	+
	7498 (Died at 14 dpi)	+	+	-	-	-	-
	7593	+	-	-	-	-	-
WT pigs housed with Challenged WT	7434 (Died at 11 dpi)	-	+	-	-	-	-
	7399 (Died at 14 dpi)	+	+	-	-	-	+
	7648 (Died at 19 dpi)	-	+	+	-	-	-
Challenged CD163E7D	81601	-	-	-	-	-	-
	81602	-	-	-	-	-	-
	81603	-	-	-	-	-	-
	82101	+	-	-	-	-	-
	82102	-	-	-	-	-	-
	82103	-	-	-	-	-	-
WT pigs housed with challenged CD163E7D	7589	+	-	-	-	-	-
	7592	+	-	-	-	-	-
	7530	++	+	++	-	-	-
	7591 (Died at 20 dpi)	-	+	++	-	-	-

^a^ “+”, “++”: PRRSV positive. ^b^ “-”: PRRSV negative.

## Abbreviations

CRISPR: Clustered regularly interspaced short palindromic repeats
DC-SIGN: dendritic cell-specific intercellular adhesion molecule-3-grabbing non-integrin
DMEM: Dulbecco's modified Eagle's medium
FBS: fetal bovine serum
DPI: day post inoculation
gRNA: guide RNA
Hb: Hemoglobin
Hp: haptoglobin
HP-PRRSV: highly pathogenic porcine reproductive and respiratory syndrome virus
HS: heparin sulphate
OTSs: off-target sites
PAM: protospacer adjacent motif
PAMs: porcine alveolar macrophages
PFFs: porcine fetal fibroblasts
PRRS: porcine reproductive and respiratory syndrome
PRRSV: porcine reproductive and respiratory syndrome virus
SCNT: somatic cell nuclear transfer
SN: sialoadhesin
SRCR: scavenger receptor cysteine-rich
WT: wild-type
