# Supplementary material for: Deletion of CD163 Exon 7 Confers Resistance to Highly Pathogenic Porcine Reproductive and Respiratory Viruses on Pigs
Source: Int J Biol Sci. 2019 Jul 25;15(9):1993–2005. doi: 10.7150/ijbs.34269 (PMC6743298; doi:10.7150/ijbs.34269)
Supplement: Supplementary file 1 — Supplementary figures and tables. [file ijbsv15p1993s1.pdf]

Table S1. Primers for off-target site of 501-sgRNA

Primers	Forward	Reverse	Product Size
Off site1	GTACAGTGTGACAGAAGCTGAAGAAG	GGCAACTTGGATCTGAACCTCG	444bp
Off site2	TGGGAACTCCTCAATGAGAACG	GGTAAAACTCAGTAAACAGGCAAGC	620bp
Off site3	ATGGAAACAGTGAGCGGCTAG	GAAGGTGGTTAAGTGTCACAGGAGC	593bp
Off site4	ATGTCATACCCTCTGAGGTCCTTC	TTGATGACCCTCCCTTCAACAGC	607bp
Off site5	AGCAGAGGCAGGAGGTATAGACA	TGATTGGGTCTCTAGCCACTGTC	545bp
Off Site6	GAATACAAAGCTAGAGGGGAACT	GGGATGTAGCAGTGAACAAGTC	600bp
Off site7	CAAAGAGCTGCACCACTGTA	CTGTACCACAACACGGAAACA	610bp

Table S2. General information of the viral challenged pigs

Groups	Pigs ID	Inoculated or Not	Virus
Challenged CD163E7D	82101; 82102; 82103; 81601; 81602; 81603	Inoculated	JXA1, $10^{6.5}$ $TCID_{50}$
WT housed with challenged CD163E7D	7589; 7592; 7530; 7260; 7331; 7591	Not Inoculated	--
Challenged WT	7345; 7352; 7473; 7482; 7498; 7593	Inoculated	JXA1, $10^{6.5}$ $TCID_{50}$
WT housed with challenged WT	7362; 7434; 7531; 7399; 7404; 7648;	Not Inoculated	--

Table S3. A detailed description of the macroscopic lesions of every experimental pig

**Groups**	**Pigs ID**	**Macroscopic lesions observed**
**Challenged CD163E7D**	81601	None
	81602	None
	81603	None
	82101	None
	82102	None
	82103	Lymph node hemorrhage
**WT housed with challenged CD163E7D**	7589	Pulmonary parenchymal lesion; lymph nodes hemorrhage; spleen infract;
	7592	Pulmonary parenchymal lesion; lymph nodes hemorrhage;
	7530	Pulmonary parenchymal lesion; lymph nodes hemorrhage; spleen infract;
	7260	Pulmonary parenchymal lesion; lymph nodes hemorrhage;
	7331	Pulmonary parenchymal lesion; lymph nodes hemorrhage; spleen infract;
	7591[a]	Lung hemorrhage; lung edema; spleen infract; kidney with blood spots; secondary bacterial infections; lymph node hemorrhage; tonsil hemorrhage;

**Challenged WT**	7345[a]	Lung hemorrhage; lung edema; spleen infract; kidney with blood spots; secondary bacterial infections; lymph node hemorrhage; tonsil hemorrhage;
	7352	Pulmonary parenchymal lesion; lymph node hemorrhage;
	7473	Pulmonary parenchymal lesion; lymph node hemorrhage; tonsil hemorrhage;
	7482	Pulmonary parenchymal lesion; lymph node hemorrhage; spleen infract; tonsil hemorrhage;
	7498[a]	Lung hemorrhage; lung edema; spleen infract; kidney with blood spots; lymph node hemorrhage; tonsil hemorrhage;
	7593	Pulmonary parenchymal lesion; lymph node hemorrhage;
**WT housed with challenged WT**	7362	Pulmonary parenchymal lesion; lymph node hemorrhage;
	7434[a]	Lung hemorrhage; lung edema; spleen infract; kidney with blood spots; lymph node hemorrhage; tonsil hemorrhage;
	7531	Pulmonary parenchymal lesion; lymph node hemorrhage;
	7399[a]	Lung hemorrhage; lung edema; spleen infract; kidney with blood spots; lymph node hemorrhage; tonsil hemorrhage;
	7404	Pulmonary parenchymal lesion; lymph node hemorrhage;
	7648[a]	Lung hemorrhage; lung edema; spleen infract; kidney with blood spots; lymph node hemorrhage; tonsil hemorrhage;

[a] The pig died before the end of the viral challenge.

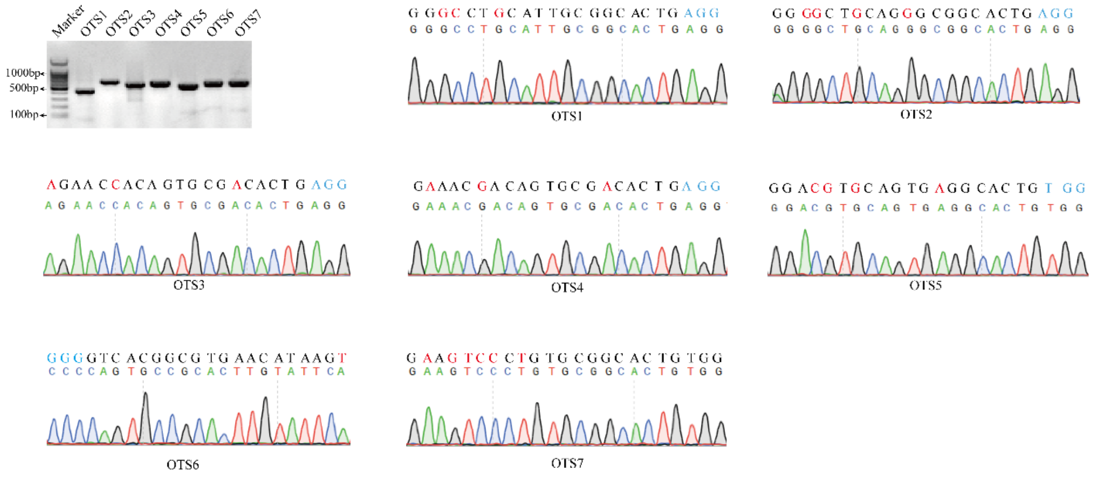

Fig. S1. Off-target analysis of colony #44. There are no off-target point mutations detected in the genome of colony #44. The 7 putative off-target sites for 501-sgRNA were amplified with PCR and sequenced. The nucleotides mismatched with 501-sgRNA were labeled red, and the PAMs were labeled blue.

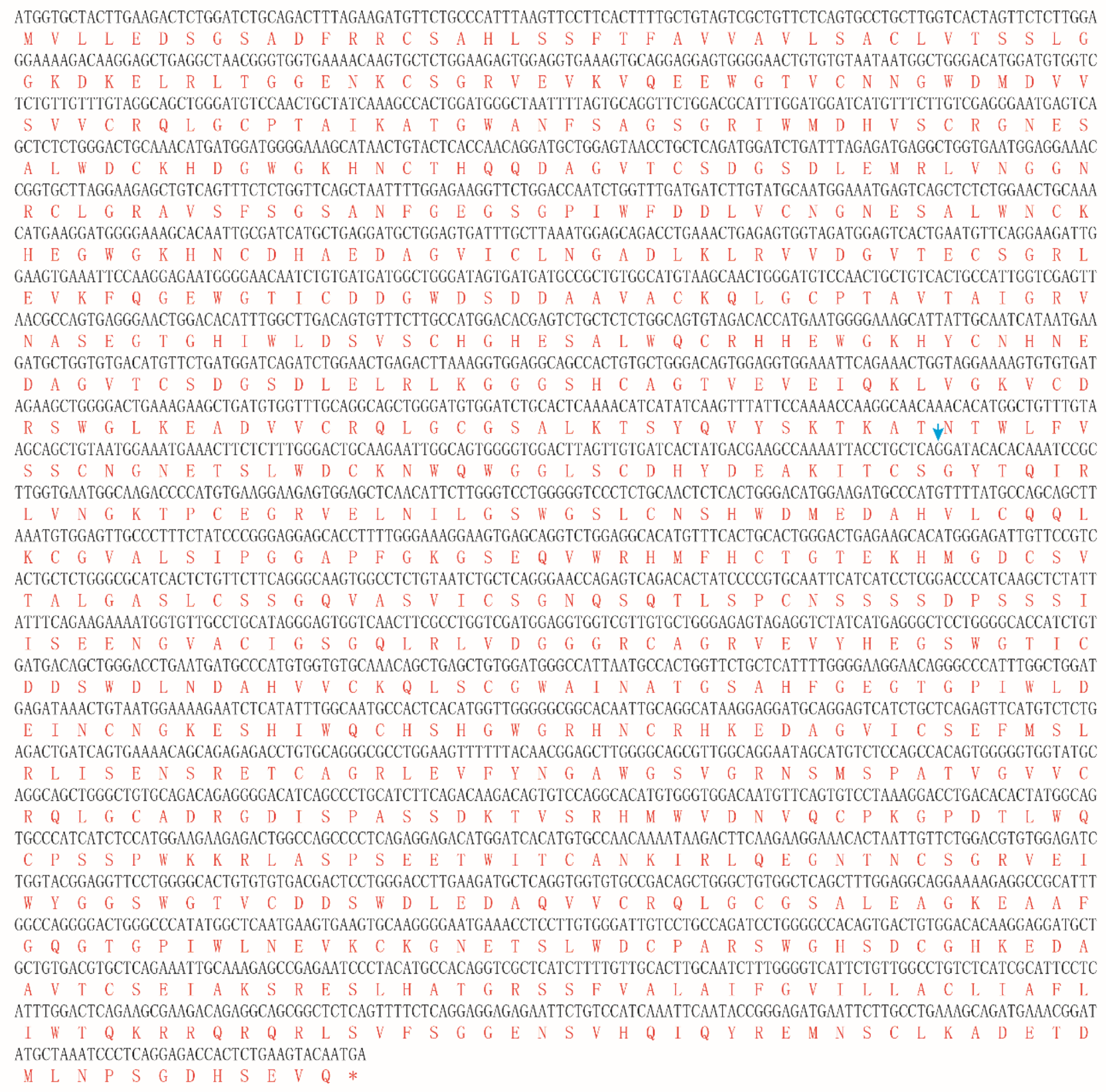

Fig. S2. *CD163* cDNA sequence and predicted amino acid sequence of the CD163E7D pigs. The cDNA sequence is 3018bp. The amino acids were labeled in red. The blue arrow indicated the position of the deleted exon 7 of *CD163*.

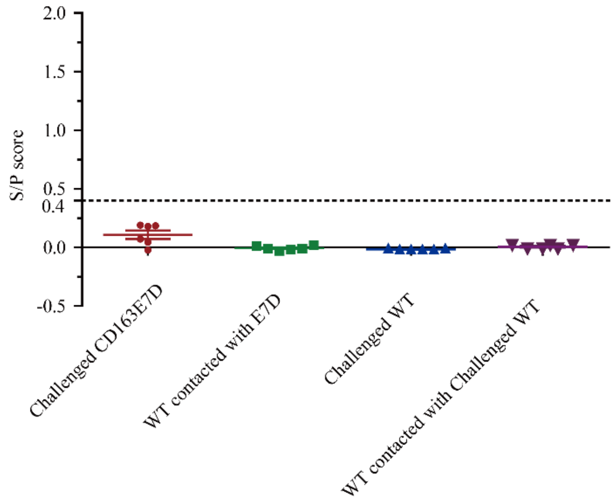

Fig. S3. The S/P scores of the challenged WT and CD163E7D pigs, as well as the pigs housed with them, proved that there was no detectable serum antibody against PRRSV nucleocapsid (N) protein in the 4 groups of pigs before the challenge.

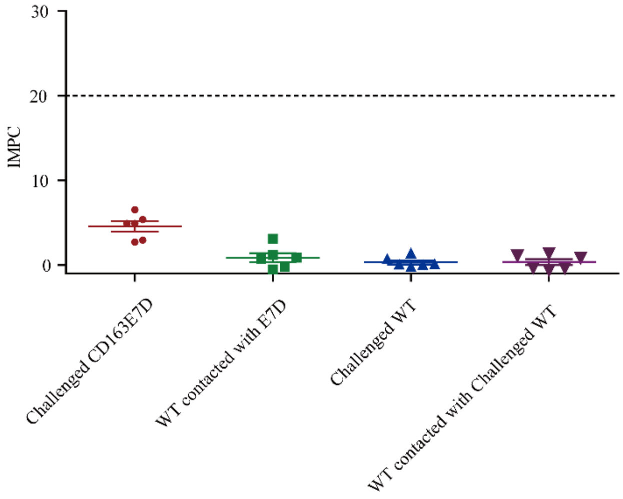

Fig. S4. There was no detectable PRRSV neutralizing antibody in the serum of the challenged WT and CD163E7D pigs, as well as the pigs housed with them, before the viral challenge.

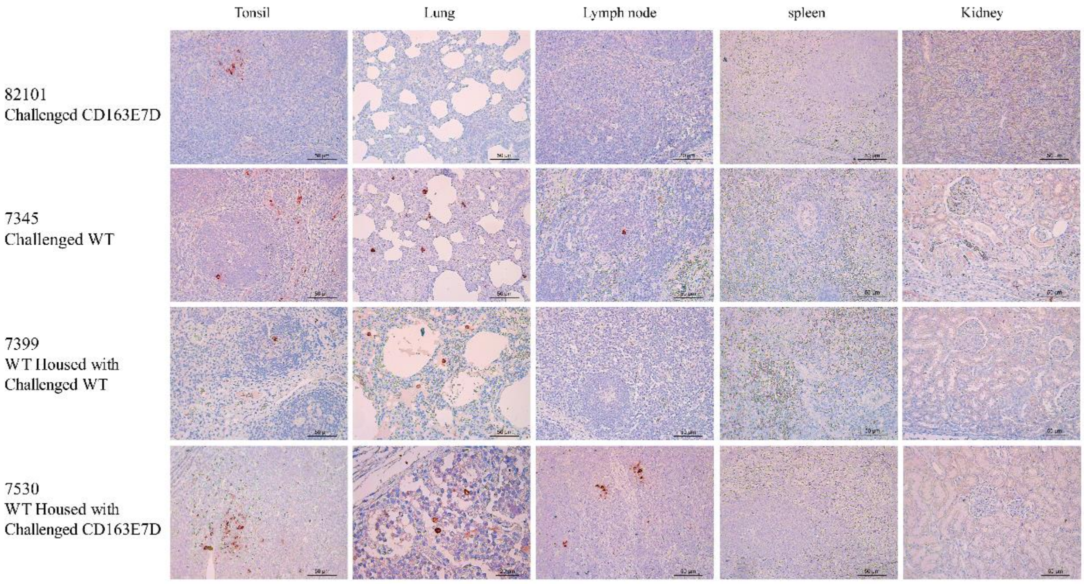

Fig. S5. Immunohistochemical staining of the lungs, spleens, kidneys, lymph nodes and tonsils of the HP-PRRSV infected pigs. PRRSV signals (red) were visualized using the primary antibody against the viral nucleocapsid protein.
